# Polymerization-Enhanced Photophysical Performances of AIEgens for Chemo/Bio-Sensing and Therapy

**DOI:** 10.3390/molecules28010078

**Published:** 2022-12-22

**Authors:** Shanshan Huang, Guogang Shan, Chao Qin, Shunjie Liu

**Affiliations:** 1 National & Local United Engineering Laboratory for Power Batteries, Department of Chemistry, Northeast Normal University, Changchun 130024, China; 2 Key Laboratory of Polymer Ecomaterials, Changchun Institute of Applied Chemistry, Chinese Academy of Sciences, Changchun 130022, China

**Keywords:** aggregation-induced emission, polymerization, photophysical performance, sensor, photosensitization, room-temperature phosphorescence

## Abstract

AIE polymers have been extensively researched in the fields of OLEDs, sensing, and cancer treatment since its first report in 2003, which have achieved numerous breakthroughs during the years. In comparison with small molecules, it can simultaneously combine the unique advantages of AIE materials and the polymer itself, to further enhance their corresponding photophysical performances. In this review, we enumerate and discuss the common construction strategies of AIE-active polymers and summarize the progress of research on polymerization enhancing luminescence, photosensitization, and room-temperature phosphorescence (RTP) with their related applications in chemo/bio-sensing and therapy. To conclude, we also discuss current challenges and prospects of the field for future development.

## 1. Introduction

Organic luminescent materials are widely used in frontier fields of optoelectronic devices [1,2], chemical sensing [3], and biological theranostics [4,5,6,7]. The development of new organic fluorophores has always been a research hotspot. However, conventional organic fluorophores usually possess planar structures and exhibit well emission behavior when dispersed in the solvent at a low concentration state. Nonetheless, in a high concentration or aggregation state, the close packing between molecules leads to strong π-π interaction and thus exhibits severe emission quenching, i.e., the ACQ effect [8,9]. The fact of only working in dilute solutions has greatly limited their practical applications [10,11,12,13]. Researchers have tried to reduce the degree of intermolecular aggregation by physical and chemical modification or engineering methods to prohibit the ACQ effect, but only minimal achievements have been obtained [14]. In 2001, Tang et al. [15] reported an opposite phenomenon, in which fluorophores emit none or weak luminescence at low concentrations, but exhibit brighter fluorescence emission at high concentrations or in aggregated states. Such fluorophores usually possess twisted molecular structures with freely rotatable or vibrating structural units, and the free motion of the molecules is restricted in the aggregated state, reducing the energy dissipation caused by molecular motion and facilitating the radiative relaxation, thus exhibiting enhanced fluorescence, i.e., the AIE effect with the most widely accepted mechanism, restriction of intramolecular motion (RIM) [9,16]. In recent years, molecular structures of AIE-active luminescent materials have been precisely controlled with different photophysical properties to meet the application demand of different fields. Tang and Lam et al. [17] have designed and synthesized a series of AIE-active E/Z isomers through the fine-tuning regulation of molecular structure. The subtle differences in structure allow them to show great differences in luminescence and biotoxicity, which makes them potential candidates in the field of visual drug screening and effect evaluation. For chemo-sensing, AIEgens are widely used as detection probes for a wide range of metal ions, which usually possess excellent luminescence efficiency and well photostability in the aggregate state to be recognized by the naked eye, and high sensitivity for precise identification of trace analytes. A systematic review has been presented by Tang et al. [18]. For the biomedical field, AIE-based bioprobe has developed vigorously, from bio-constituent sensing to imaging and diagnostic therapeutics, due to their good photostability and photobleaching resistance, higher signal-to-noise ratio, and sensitivity, etc., as summarized in depth by Tian et al. [19].

**Figure 1:**
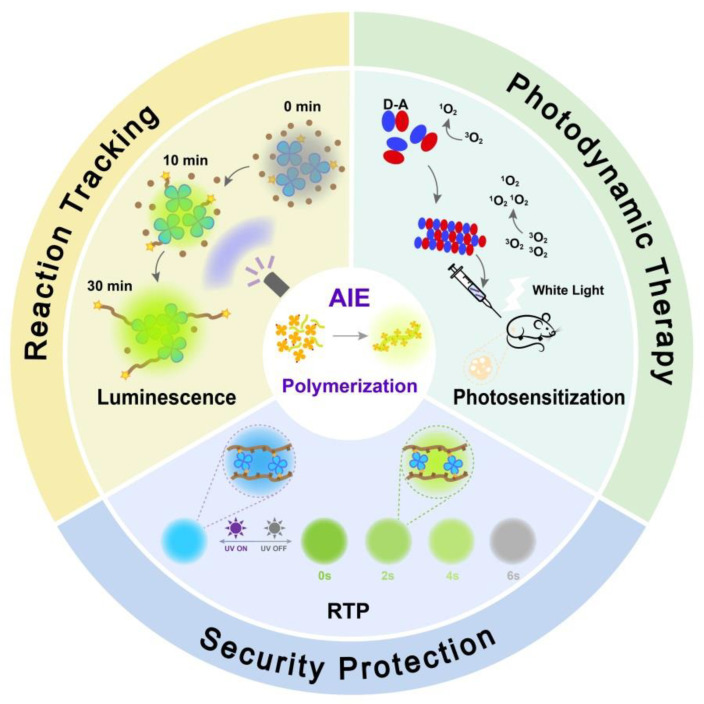
The schematic diagram of the representative polymerization-enhanced photophysical properties and applications.

From the perspective of materials, most of the current research is concentrated in the field of small molecules with more controllable structures, and the development and application of fluorescence polymers still have a lot of room for exploration and enhancement. Polymer materials have gradually become a research hotspot in various fields due to their high thermal stability, good ductility and other advantages combined with luminescent properties and information about fluorescence polymers with different characteristics and applications have been summarized in detail by these recent reviews [20,21,22]. The Introduction of AIE fluorophore units into the structure of polymer side chains or backbone could appropriately tune its morphology and composition to obtain well-defined novel functional materials with multiple advantages such as high brightness, stability, and biocompatibility. Since the first AIE polymer was designed and prepared by Tang et al. [23] in 2003, the preparation and application of AIE-active polymers have been continuously expanded. MOF (metal-organic framework) and COF (covalent organic framework) are also common polymeric materials. Li et al. [24] prepared a highly stable yellow-emitting MOF, LMOF-231 by immobilizing the AIE-active chromophore H_4_tcbpe into a rigid framework structure and was coated on the surface of commercial blue LED for the successful preparation of white-emitting LED. In addition, AIE-active fluorophore of novel organic-inorganic hybrid compounds with unique luminescent characteristics constructed by cheap and non-toxic metal ions such as d^10^ zinc cations has also been a new research hotspot [25]. In recent years, as shown in Figure 1, researchers have found that some photophysical properties such as luminescence intensity, as well as photosensitization ability, two-photon absorption intensity, etc. can be significantly improved and enhanced by polymerization strategies on the basis of small molecules [26,27,28]. For example, the enlarged conjugation of conjugated polymers allows for enhanced light-trapping capabilities compared to small-molecule model compounds [29]. At the same time, with the gradual increase of the conjugation degree, the Δ*E*_ST_ value (energy gap between the singlet and triplet states) of the polymer gradually decreased, which facilitated the process of ISC (intersystem crossing) and thus effectively enhanced the generation efficiency of ROS [30]. In addition, the polymerization strategy also has certain applications in inducing the production of RTP [31], TADF [32], and so on.

In this review, we discussed the common synthesis strategies of AIE-active polymers and enumerated the phenomenon, mechanisms, and effects where significant improvements in photophysical properties have been obtained by polymerization, and briefly discussed the opportunities and challenges for further development.

## 2. Design of AIE-Active Polymers

**Figure 2:**
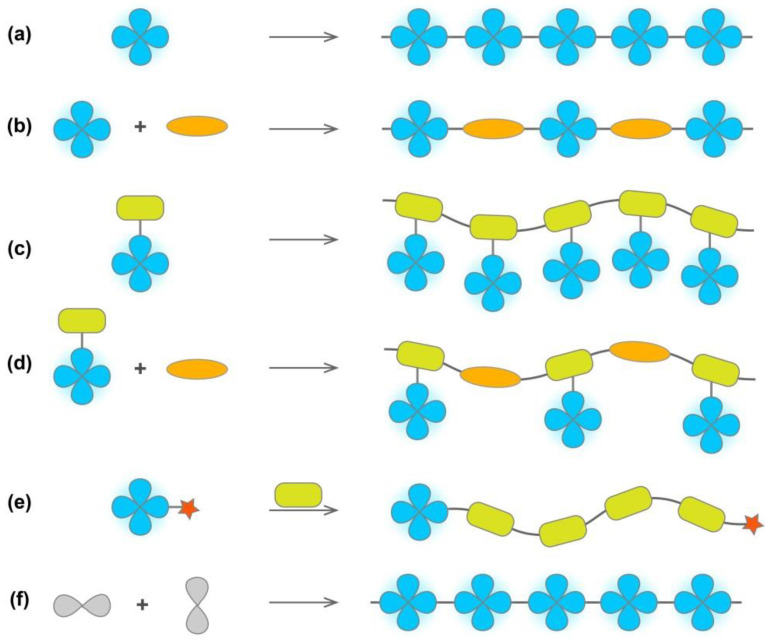
Construction strategies of AIE-active polymers. (**a**) Direct polymerization of AIE-active monomers and (**b**) copolymerization of AIE-active monomers with other monomers to embed AIEgens into the main chain of polymer. (**c**) Homopolymerization and (**d**) copolymerization to introduce AIEgens into the side chain of polymer. (**e**) AIEgens served as an initiator to initiate polymerization. (**f**) Polymerization of AIE-inactive precursors to obtain polymers with AIE property.

Compared with small molecular systems, polymer materials still have a lot of space for development in terms of morphology regulation and structural modification. Therefore, it is of great significance to develop mild, efficient, and environmentally friendly polymerization strategies to construct novel functional AIE-active polymer materials. In general, AIE-active polymer can be designed and prepared through various polymerization, the basic principle of which is usually the introduction of AIEgens into the main or side chain of the polymer by means of insertion, linking, or grafting. Figure 2 illustrates several basic approaches to embed AIEgens into the polymer main chain by (a) direct polymerization of AIE-active monomers or (b) copolymerization with other monomers. Similarly, the attachment of AIEgens to polymerizable monomers, followed by homopolymerization (c) or copolymerization (d) reactions can introduce them into the side chains of the liner polymer. In addition, AIEgens served as an initiator to initiate polymerization (e) is also a common method to obtain AIE-active polymers. Alternatively, AIE-inactive precursors can also be polymerized to obtain polymers with AIE property (f). More details are summarized by other reviews [14,33,34,35,36].

## 3. Polymerization-Enhanced Luminescence for Reaction Tracking and Responsive Materials

**Figure 3:**
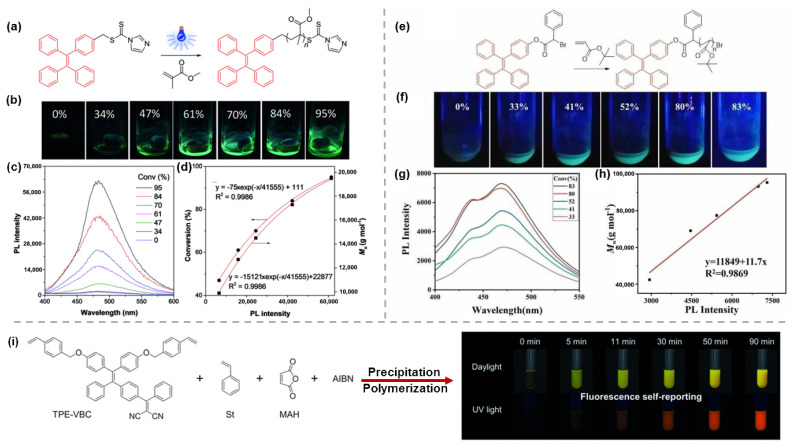
(**a**) Illustration of the RAFT reaction process. (**b**) The fluorescent photographs of the polymer solutions at different conversions taken under UV irradiation. (**c**) PL spectra of the polymerization mixtures at different conversions. (**d**) The exponential relationship of conversion and Mn with PL intensity. Reprinted with permission from Ref. [38]. 2018, Wiley-VCH. (**e**) Illustration of the ATRP reaction process. (**f**) Fluorescent photographs of the polymer solutions at different conversions taken under UV irradiation. (**g**) PL spectra of the polymerization mixtures at different conversion. (**h**) The linear relationship of Mn with PL intensity. Reprinted with permission from Ref. [40]. 2020, Wiley-VCH. (**i**) Illustration of the precipitation polymerization process and fluorescent photographs of the mixtures monitored under daylight and UV light at different times. Reprinted with permission from Ref. [41]. 2019, Wiley-VCH.

During the reaction, the physical properties of the polymerization system, such as luminescent color, viscosity, solubility, etc., usually change continuously over time, so it is of great importance to design and prepare high-performance polymers by effectively monitoring the degree and process of the reaction through fluorescence techniques to achieve visualized controlled polymerization [37]. Tang et al. [38] combined photochemistry with AIE technology to achieve a high degree of visualization of the reversible addition fragmentation chain transfer (RAFT) polymerization process without destroying the reaction system. The quenching effect caused by the carbonyl sulfur groups resulted in the non-luminescence of TPE-based RAFT agents in either solid or liquid states [37,39]. As the reaction proceeded, the obtained polymer exhibited strong luminescence with AIE properties. As shown in Figure 3b, as the conversion rate of below 34%, the system was barely emissive. When gradually increased to 84%, the luminescence was rapidly enhanced due to the increase in system viscosity. Finally, as the conversion rate was higher than 84%, the viscosity of the system was almost unchanged, and the emission intensity increased slowly and tend to be stable. The PL spectra presented in Figure 3c,d showed the same trend, and the resulting polymer PMMA showed an exponential increase in Mn over fluorescence intensity. Inspired by this, Pang et al. [40] reported another similar work in which TPE-3, exhibiting the highest polymerization conversion, was applied as an AIE-active initiator to carry out atom transfer radical polymerization (ATRP) with *t*BA (*tert*-butyl acrylate), a commonly used monomer. As time went by, the fluorescence intensity of the system increased gradually, and as the conversion rate reached 80%, the increase in emission intensity tended to be gentle (Figure 3f). Meanwhile, as shown in Figure 3h, a linear relationship between the PL intensity of the system and Mn was achieved, which enabled a non-invasive visualization of the polymerization process of the ATRP system.

**Figure 4:**
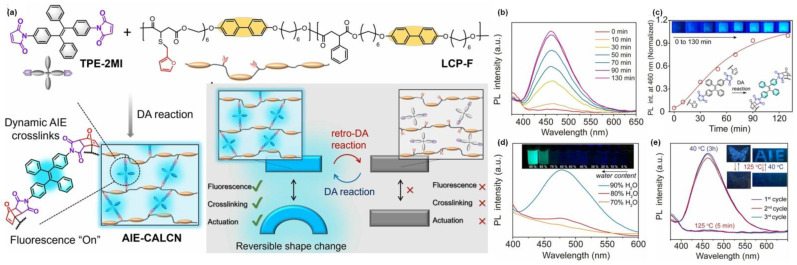
(**a**) The chemical structures and dynamic mechanism of the prepared AIE-CALCNs via the reversible of DA reaction. (**b**) PL spectra of TPE-2MI/LCP-F film keeping at 40 °C for different durations. (**c**) Plot of fluorescence intensity versus different times at 40 °C, and photographs of the sample taken under UV light. (**d**) PL spectra and photographs of AIE properties of TPE-2MI-furan reactants. (**e**) PL spectra and photographs of AIE-CALCN over multiple cycles of switching taken under UV light. Reprinted with permission from Ref. [42]. 2022, Wiley-VCH.

In addition to the above two polymerization reactions, precipitation polymerization was also a common means of polymerization. Tang et al. [41] used AIBN as initiator and AIE-active TPE-VBC, styrene (St), and maleic anhydride (MAH) as monomers to carry out the polymerization in isopentyl acetate (IAAC) and monitored the reaction under sunlight and UV light for different times. As the AIE-active TPE-VBC was grafted onto the polymer backbone, the conformation of the molecular backbone changed with the progress of the reaction and gradually confined to particles, leading to the changes in emission intensity and the visualization of reaction microenvironments. As shown in Figure 3i, for the first 5 min, although the color of the system changed in daylight, the fluorescence of the system was still weak under UV light, which was probably due to the low degree of polymerization at this time. With the passage of time, the degree of polymerization gradually increased, and the intermolecular interactions were also enhanced, resulting in a bright orange emission under UV light. When increased to the critical point, the solubility of the system dropped sharply, and the obtained polymer began to aggregate and phase-separate gradually. Recently, Zhao et al. [42] prepared covalent adaptable liquid crystal networks with AIE properties by using the tetraphenylene derivative TPE-2MI as a fluorescent probe and dynamic cross-linking agent, and reacted with LCPF via thermally reversible Diels-Alder (DA) reaction to enable visual monitoring of network dynamics. As shown in Figure 4b,c, the initial phase of the mixture was non-emissive because of the quenching effect caused by the photo-induced electron transfer (PET) mechanism. As the reaction proceeded at 40 °C, DA adducts were formed on both sides of TPE-2MI, which eliminated the PET process, and the blue-green fluorescence of the system gradually increased. At the same time, furan and TPE-2MI was selected as model molecules to observe that TPE-2MI-furan DA adducts exhibited obvious AIE characteristics with increasing water content (Figure 4d), indicating that the AIE effect was also responsible for the enhanced fluorescence of the reaction system. Due to the reversibility of DA reaction, when the system is heated at 125 °C for 5 min, the DA bond dissociated, accompanied by the disappearance of fluorescence in the system, followed by holding at 40 °C for 3 h, the DA bond restored with the fluorescence recovered again. The process can be repeated for multiple cycles without any significant attenuation of fluorescence intensity, and almost completely recovered, which indicated the well thermal regulation stability of the prepared AIE-CALCNs. Meanwhile, based on the above results, a good correlation between the cross-linking state of DA reaction system and the fluorescence emission intensity was achieved, which makes the information of cross-linking state of AIE-CALCN material visually displayed by fluorescence signal. As a demonstration, (Figure 4e) the pattern drawn on LCP-F film with TPE-2MI/ acetone solution can be reversibly erased and revealed many times under different conditions (heating at 125 °C for 5 min or keeping at 40 °C).

**Figure 5:**
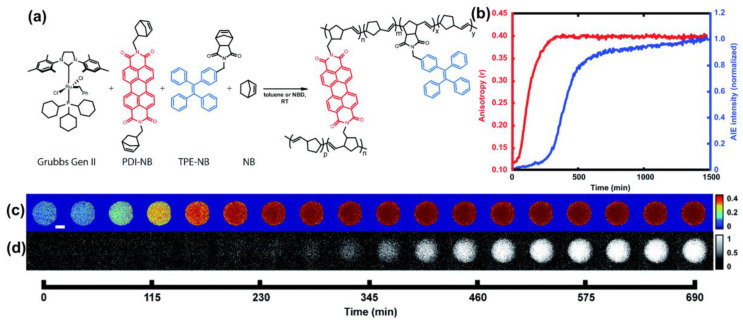
(**a**) Illustration of the ROMP reaction process. (**b**) The relationship of average anisotropy and AIE intensity with time. (**c**) The photographs of anisotropy values in droplets over time. (**d**) Emission intensity of the AIE monomer probe in a droplet over time. Reprinted with permission from open access of Ref. [43]. 2020, Royal Society of Chemistry.

Visualized polymerization based on AIE mechanism could also be combined with other detection methods, so as to control the degree of polymerization in a more precise way. Based on fluorescence polarization anisotropy, the measurement of fluorophore rotational dynamics to obtain changes in the chemical environments in the reaction has been widely used in a variety of polymerization systems. Goldsmith et al. [43] applied fluorescence anisotropy for the first time to monitor the chemical reaction progress in droplets, combining AIE effect with the introduction of TPE-NB monomers with AIE properties to jointly track the reaction process of the polymerization system. Polymerization reactants and conditions were shown in Figure 5a, Ruthenium-based Grubbs Generation II-catalyzed ring-opening metathesis polymerization (ROMP) were selected, TPE and PDI-based norbornene monomers were selected as fluorescent probes, and the reaction-prepared droplet arrays were imaged using fluorescence microscopy. As shown in Figure 5c, the anisotropy of the mixture increases rapidly during the initial stage of the reaction, but soon plateaus. At this point, the AIE measurements complemented the reaction process well, with a late onset of the AIE response over time, continued to increase after the anisotropy measurements reached their highest value, and was able to be monitored continuously for up to several hours (Figure 5d). The results suggest that fluorescence polarization anisotropy may be more suitable for efficiently judging whether the polymerization reaction has occurred in the system, while subsequent reaction processes, such as whether a larger Mn has been reached, can be detected by AIE measurement. Such a combination of two complementary approaches provided new ideas for the design of monitoring polymerization processes with fluorescence self-reporting properties.

**Figure 6:**
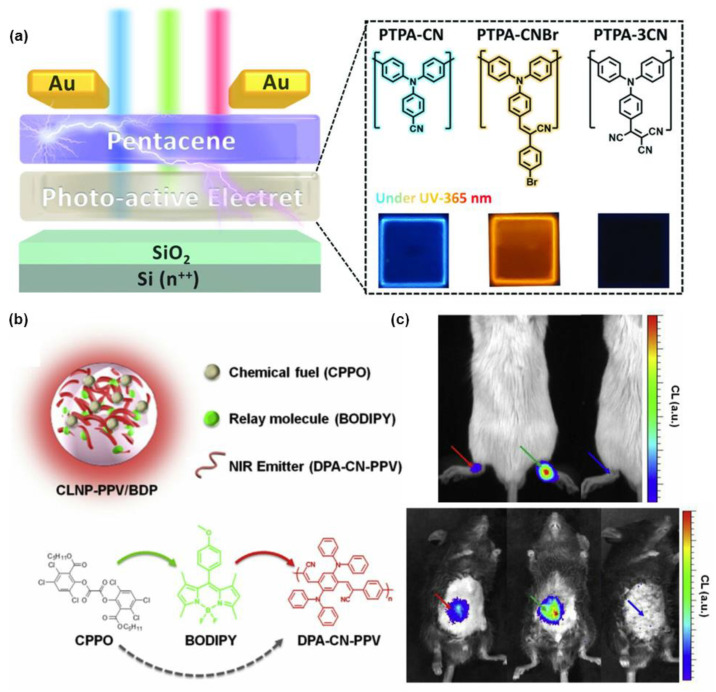
(**a**) The chemical structures of the relevant molecular and schematic illustration of photoprogrammable recorder device. Reprinted with permission from Ref. [44]. 2021, Wiley-VCH. (**b**) Schematic illustration of the structure of energy-relayed POCL nanoparticle (CLNP-PPV/BDP) and corresponding molecules. (**c**) The photographs of mouse model of arthritis and peritonitis. Reprinted with permission from Ref. [45]. 2016, Elsevier.

In addition to reaction tracking, AIE polymers have also been extensively studied in the field of responsive materials. Recently, Liou et al. [44] designed the AIE-active conjugated polymer with fine-tuned DA structure as an electret for high-performance optical programmable memory with electrical writing/photoerasing functions (Figure 6a). The device has an ultra-fast optical response time (0.1 ms), excellent current switching ratio (106), and ultra-high stability (hold time up to 40,000 s). Meanwhile, different storage behaviors can be switched from flash to WORM by regulating the torsion angle between the donor and acceptor structural parts, which provides a new idea for the design of ultra-fast optical storage device materials.

In addition to chemical sensing, functional polymers also have applications in biosensing. Kim et al. [45] designed an efficient signal-boosted nanophotonic probe (CLNP-PPV/BDP) as an “energy relay” system. As shown in Figure 6b, peroxide CPPO was served as “chemical fuel” and green luminescent BODIPY as the “relay molecule”. The low band gap near-infrared AIE conjugated polymer DPA-CN-PPV with unique photonic characteristics is effectively excited through the “energy relay” of this BODIPY molecule, achieving intracellular and in vivo NIR imaging of H_2_O_2_, with a low detection limit of 10^−9^ M and a tissue penetration depth over 12 mm, which makes it possible to deeply image inflammatory H_2_O_2_ in mice (Figure 6c).

## 4. Polymerization-Enhanced Photosensitization for Photodynamic Therapy and Photocatalysis

**Figure 7:**
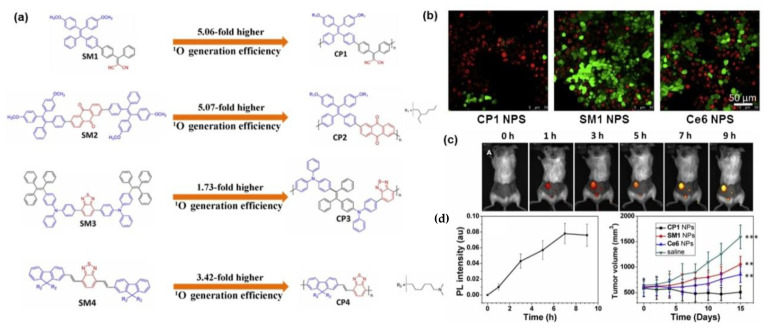
(**a**) The comparison of the working mechanism of photosensitizers between small molecules and conjugated polymers. (**b**) The chemical structures of small molecules SM1–SM4 and conjugated polymers CP1–CP4 with enhanced ^1^O_2_ generation efficiency. (**c**) Comparative photographs of the effects of PDT based on CP1 NPS, SM1 NPS, and Ce6 NPS at the cellular level. (**d**) Photographs of CP1, SM1, and Ce6 NPs for in vivo image-guided PDT. *** *p* < 0.001, ** *p* < 0.01. Reprinted with permission from Ref. [30]. 2018, Elsevier.

Compared to traditional clinical treatments, photodynamic therapy (PDT) has shown great application prospects in the therapy of a wide range of diseases due to its advantages of non-invasiveness, strong controllability, and few side effects. Photosensitizers, together with oxygen and light sources, were called the three essential elements of PDT. Common photosensitizers mainly include phthalocyanine [46,47], porphyrin [48], and BODIPY derivatives [49]. In recent years, the design and synthesis of small molecular photosensitizers have developed rapidly. Tang et al. [50] reported a dual-functional photosensitizer, which showed an ultra-high ^1^O_2_ quantum yield of up to 98.6% in the aqueous solution and could simultaneously exhibit excellent PDT effects and real-time monitoring of treatment in in vivo. Dong et al. [49] designed a D-A-D structure photosensitizer, DPPBDPI, using Diketopyrrolopyrrole (DPP) and BODIPY as building units with ^1^O_2_ quantum yield of over 80%, showing good PDT capabilities at both in vitro and in vivo. How to achieve more efficient ROS yield has always been the core content of photosensitizer design. The corresponding design strategies and classification of photosensitizers based on small molecules have been reviewed in detail by the groups of Prof. Peng [51] and Prof. Li [52]. Recently, polymerization-enhanced photosensitization has also been proven to be one of the most effective methods to improve the efficiency of ROS generation [30,53,54]. Compared with small molecule photosensitizers, the extended conjugation length endowed conjugated polymers with greater enhancement of light capture ability [30,55,56,57]. Meanwhile, AIE effect also solved the problem of reduced ROS yield due to decreased fluorescence intensity caused by the ACQ effect. Nowadays, AIE-active conjugated polymer photosensitizers have been extensively studied in the field of photodynamic therapy [58,59,60]. Liu et al. [30] selected four reported small-molecule photosensitizers SM1-SM4 and their corresponding conjugated polymers CP1-CP4 after polymerization (Figure 7a) for comparing their ^1^O_2_ generation efficiency. The results indicated that the ^1^O_2_ generation efficiency of the obtained conjugated polymers has increased by 5.06, 5.07, 1.73, and 3.42 times, respectively, compared with their model compounds. Time-dependent density functional theory (TD-DFT) calculations have shown that the increase of repeating conjugated units have reduced the difference between the upper excited states (S_n_ and T_n_) energy levels and the lowest excited states (S_1_ and T_1_) [61], which promoted the ISC process and thus the yield of singlet oxygen was effectively enhanced. On the other hand, the molar absorption coefficient of the polymer was enhanced with the increase of the polymer repeating units, and the enhanced light trapping ability also promoted the photosensitization effect of the polymer. Among them, CP1 showed the highest single-linear oxygen generation efficiency, which was 3.71 times higher than that of the commonly used commercial photosensitizer dye Ce6. The corresponding nanoparticles were prepared by encapsulating SM1, CP1, and Ce6 with DSPE-PEG2000 as a polymer matrix, respectively, and used for in cell and in vivo photo-induced cancer cell ablation and tumor therapy, with all the results showing that CP1 NPs exhibited significant better PDT efficiency (Figure 7b,c).

**Figure 8:**
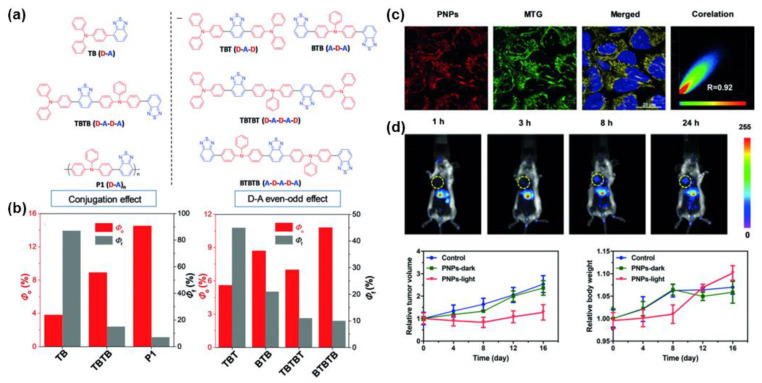
(**a**) The chemical structures of TB, TBTB, P1, TBT, BTB, TBTBT, and BTBTB. (**b**) ^1^O_2_ quantum yield (*Φ*_o_) and fluorescence quantum yield (*Φ*_f_) of the above materials. (**c**) Colocalization fluorescence image of HeLa cells. (**d**) Tumor imaging of mice and in vivo PDT effects of PNPs. Reprinted with permission from Ref. [54]. 2018, Wiley-VCH.

On this basis, Tang et al. [54] proposed that D-A even–odd effect is another strategy to enhance photosensitization. As shown in Figure 8b, the ^1^O_2_ quantum yield from small molecule TB to dimer TBTB to polymer P1 existing five repeating TB units, increased from 3.8% to 8.9% and then to 14% with increasing degree of conjugation, respectively, indicating that increasing the degree of conjugation by polymerization is indeed an effective way to enhance the quantum yield of ^1^O_2_. In addition, the ^1^O_2_ quantum yield of BTB (A-D-A) with different donor-acceptor units was higher than that of TBT (D-A-D) (8.7% for BTB and 5.6% for TBT), and their corresponding conjugated polymers BTBTB (A-D-A-D-A) and TBTBT (D-A-D-A-D) showed the same trend (10.8% for BTBTB and 7.0% for TBTBT). Time-dependent density functional theory (TD-DFT) calculations revealed that the *∆E*_ST_ values of BTB (0.38 eV) and BTBTB (0.24 eV) for which the number of A unit is greater than that of D unit are lower than those of TBT (0.49 eV) and TBTBT (0.41 eV) with more D units than A units. The reduction in ∆*E*_ST_ promotes the ISC process, and thereby improving the ^1^O_2_ quantum yield, which is the so-called “D-A even–odd effect”. Later, P1 was encapsulated into water-soluble nanoparticles, PNPs, with amphiphilic DSPE-PEG_2000_ and applied for in vivo PDT on tumors. PNPs possessed a high specificity for mitochondria and exhibited well biocompatibility in the dark environment, which indicated broad application prospects in image-guided photodynamic anticancer therapy (Figure 8c,d).

**Figure 9:**
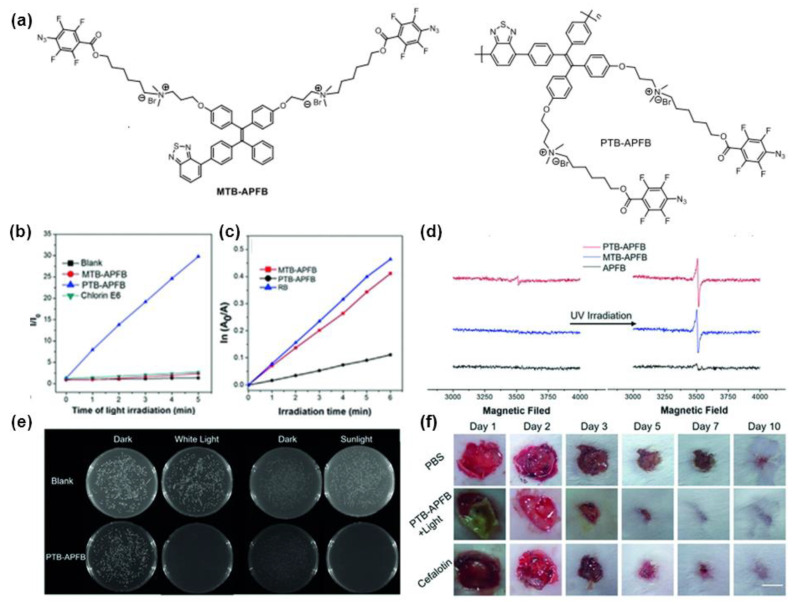
(**a**) The chemical structures of MTB-APFB and PTB-APFB. (**b**) The ROS generation ability of PTB-APFB, MTB-APFB and Ce6 upon exposure to white light for different irradiation times. (**c**) The ^1^O_2_ generation ability of PTB-APFB, MTB-APFB and RB in PBS buffer under different irradiation times. (**d**) The radical generation ability of PTB-APFB and MTB-APFB after UV irradiation. (**e**) Photographs of biocidal activity of PTB-APFB treated *S. aureus* under white light, sunlight and dark conditions. (**f**) Photographs of the mice skin infected with *S. aureus* during treatment with different formulations. Reprinted with permission from Ref. [62]. 2020, Wiley-VCH.

Profited by the enhanced light-trapping ability of the conjugated polymers, Tang et al. [62] reported another AIE-active conjugated polymer, PTB-APFB, with D-π-A structure and higher ROS production capacity in the aggregated state compared to the corresponding low-mass model compound MTB-APFB (^1^O_2_ quantum yield 38% for PTB-APFB, 29% for MTBAPFB). For in vitro and in vivo investigations, PTB-APFB can effectively suppress the infection of *S. aureus*, and the treatment recovery is faster than that of cefotaxime (Figure 9e,f), indicating great prospects for practical applications in antibacterial infections. Benefiting from these strategies, photosensitizers with high ROS generation efficiency designed by the polymerization and extended conjugation enhanced photosensitization effect have been widely used in PDT anticancer and antibacterial applications [26,60,63,64,65].

**Figure 10:**
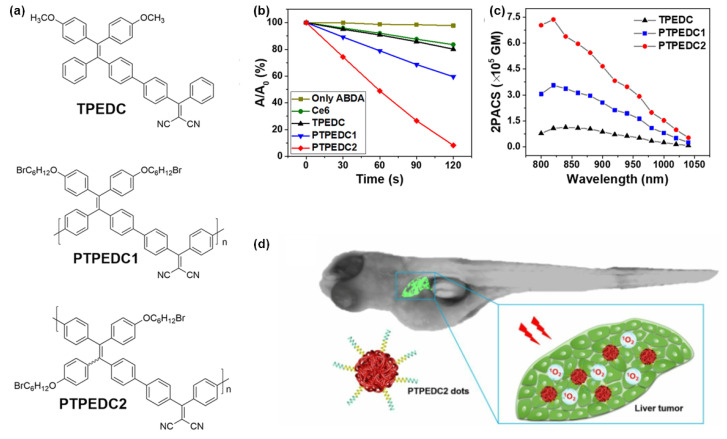
(**a**) The chemical structures of TPEDC, PTPEDC1, and PTPEDC2. (**b**) The ^1^O_2_ generation ability of TPEDC, PTPEDC1, PTPEDC2, and Ce6 upon exposure to white light for different irradiation times. (**c**) Two-photon absorption cross section (2PACS) spectra of the three materials in aqueous solution. (**d**) 2PE-PDT performance for in vivo zebrafish liver tumor treatment. Reprinted with the permission from Ref. [29]. 2019, American Chemical Society.

Furthermore, the penetration depth of tissue also has a great influence on the PDT effect. Two-photon excited photodynamic therapy (2PE-PDT) has attracted widespread attention due to its enhanced tissue penetration ability and precision. The two-photon absorption (2PA) cross-section and ^1^O_2_ generation efficiency are the so-called two important factors affecting the effectiveness of 2PE-PDT [66,67,68]. However, the high ^1^O_2_ generation efficiency means the requirement of the twisted and low-conjugated donor-acceptor structure of the photosensitizer to effectively reduce the ∆*E*_ST_ value, but a good conjugation structure is also indispensable to obtain the large (2PA) cross-section [69]. As expected, polymerization can not only enhance the photosensitization ability as mentioned above but also effectively extend the conjugation extent, providing an effective idea for the design of high-performance two-photon PSs [70]. Liu et al. [29] prepared two AIE-active polymers PTPEDC1 and PTPEDC2 with successively increased conjugation lengths based on a small molecule photosensitizer, TPEDC (Figure 10a), and the generation efficiency of ^1^O_2_ was enhanced by 2.27 and 5.48 times under white light irradiation, along with 3.15 and 6.15 times enhanced of (2PA) cross section, respectively. The three photosensitizers were encapsulated with DSPE-PEG-MAL and modified with TAT-SH to obtain water-soluble nanoparticles for further PDT experiments. As shown in Figure 10d, PTPEDC2-TAT dots not only showed the best ablation of HeLa cells in the selected precise area of 400 mm×400 mm but also achieved effective treatment of liver tumors in zebrafish, which provided an excellent idea for the design of efficient photosensitizers for 2PE-PDT.

**Figure 11:**
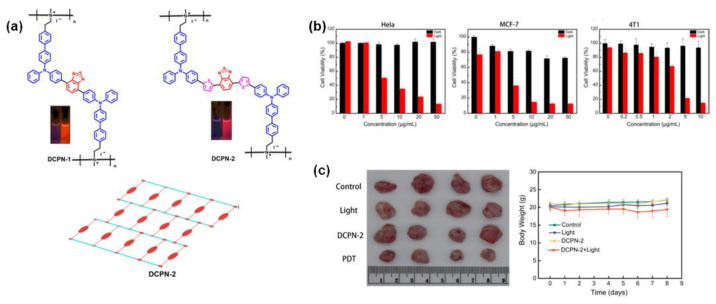
(**a**) The chemical structures of DCPN-1 and DCPN-2. (**b**) The PDT effects based on DCPN-2 in Hela, MCF-7, and 4T1 cells. (**c**) Photographs of PDT effects for tumors treated with different formulations. Reprinted with permission from Ref. [71]. 2022, Elsevier.

Following the above strategies, recently, Song et al. [71] designed and synthesized two AIE ionic polymers DCPN-1 and DCPN-2 with a reticular structure by ring-opening polymerization to favorably restrict intramolecular motions. Under white light irradiation, compared with DCPN-1, DCPN-2 exhibited stronger ^1^O_2_ production ability and exhibited favorable photodynamic therapeutic effects on the growth of MCF-7, HeLa, and 4T1 cells in vitro (Figure 11b). For the in vivo experiments, as shown in Figure 11c, the average tumor weight of the mice in “DCPN-2 + light” group decreased by 55%, and no obvious variations of the body weight were observed compared with other control groups, indicating its good PDT destructive effect on primary tumors with less toxic side effects. This work has provided a new idea for the design of biocompatible polymers with high ^1^O_2_ production ability for preclinical research and clinical applications. 

**Figure 12:**
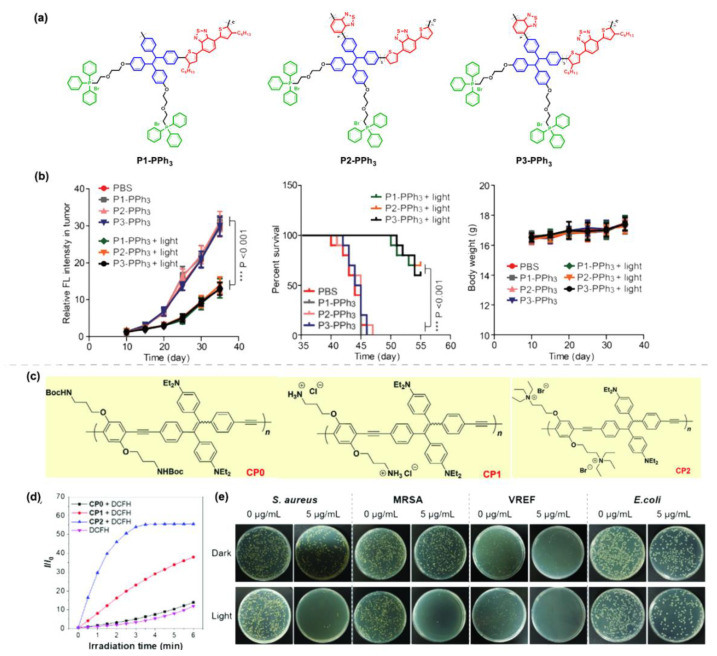
(**a**) The chemical structures of P1-PPh_3_, P2-PPh_3_ and P3-PPh_3_. (**b**) In vivo PDT effects based on the three materials. Reprinted with permission from Ref. [63]. 2020, Springer Nature. (**c**) The chemical structures of CP0-CP2. (**d**) The ROS generation ability of the three polymers. (**e**) Photographs of biocidal activity of CP2 treated *S. aureus*, MRSA, VREF, and *E. coli* in darkness or upon white light irradiation. Reprinted with permission from Ref. [72]. 2021, Wiley-VCH.

Zhao et al. [63] reported three TPE-containing red AIE-active polymers, P1-PPh3, P2-PPh3, and P3-PPh3, all of which can selectively target lysosomes with good intracellular retention ability to undergo ultra-long term tracking performance against subcutaneous tumors up to 20 days. Importantly, both in vivo and in vitro experiments showed that these polymers possessed good photostability and ^1^O_2_ production ability, effectively prolonged the survival of tumor-bearing mice by inhibiting the growth of subcutaneous tumors (Figure 12b). Tang et al. [72] introduced TPE into polymer PPE (Poly(phenyleneethynylene)) with different modifications of alkyl side chains to obtain a series of AIE-active PPE derivatives CP0-CP2 with different functions (Figure 12c). Their ROS generation ability was evaluated using DCFH (2,7-dichlorodihydrofluorescein) as a probe, the results showed that subtle changes in the side chain groups would exert an important effect on the ROS generation ability, and thus CP1 and CP2 could act as photosensitizers generating destructive ROS with potential applications in photodynamic therapy for killing multiple kinds of bacteria. In order to test this idea, the killing efficiencies of CP1 and CP2 against four bacteria, including *S. aureus* (G(+)), *E coli* (G(−)), methicillin-resistant *S. aureus* (MRSA), and vancomycin-resistant *Enterococcus faecium* (VREF) under darkness or light irradiations were explored. The results were shown in Figure 12e, CP2 exhibited a certain of dark toxicity against all four bacteria, while the killing efficiency of *S. aureus* (G(+)), drugresistant MRSA bacteria and VREF bacteria reached to almost 99% when incubated with a low concentration of 5 μg mL^−1^ after 10 min of white light irradiation, and the survival rate of *E. coli* was reduced to 22%. All the above results have demonstrated the efficient photodynamic effect of CP2 on killing Gram-positive and drug-resistant bacteria.

In addition to fluorescent materials, organic long afterglow materials have been extensively studied in various fields [73,74,75,76,77]. By profiting from their long lifetime and low toxicity, they can also be used as photosensitizers for PDT. In 2020, He et al. [78] designed and synthesized a series of ultralong organic phosphorescent (UOP) materials, EDCz (E = O, S, Se, and Te). Among them, SeDCz nanocrystals benefited from its long-lived triplet excited state, which produced ^1^O_2_ under white light irradiation, enabling the afterglow imaging and PDT of *S. aureus* for the first time, and thus providing a new strategy for the design of novel efficient photosensitizers based on UOP materials.

**Figure 13:**
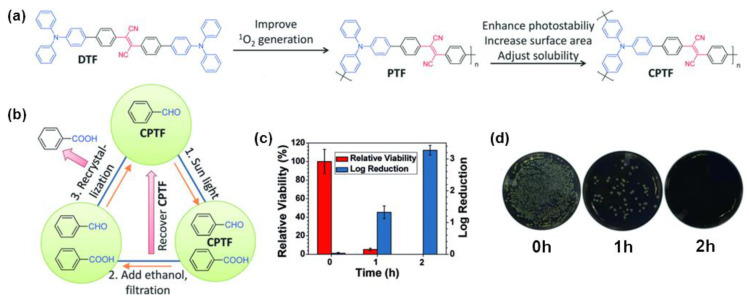
(**a**) The chemical structures of DTF, PTF, and CPTF. (**b**) The recycling process of sunlight-induced photooxidation of benzaldehyde. (**c**) The antibacterial activity of CPTF under different irradiation times. (**d**) Photographs of biocidal activity of CPTF-treated *S. aureus* upon different irradiation times. Reprinted with permission from Ref. [83]. 2019, Wiley-VCH.

In addition to phototherapy, photosensitizers are also widely used in photocatalysis, such as catalyzing organic synthesis [79,80,81], sunlight-induced wastewater treatment [82], etc. However, many photosensitizers may themselves react with the generated ROS, with reduced photostability, and were prone to photobleaching under high power irradiation, which greatly limits their application range. As mentioned above, polymerization can effectively improve the photostability of small molecule dyes and improve their photosensitization capability. Motivated by these observations, Wu et al. [83] prepared photostable conjugated polymeric PS with high ^1^O_2_ generation efficiency by a three-step design strategy. Firstly, the AIE-active DTF with a D-A structure was selected as the model molecule (Figure 13a). The introduction of the benzene ring in the molecule helps the separation of HOMO-LUMO distribution, promotes the process of intersystem crossing (ISC), and then improves the production efficiency of ^1^O_2_ [84,85]. Later the conjugated polymer PTF with the same components as DTF was further prepared, the elongated conjugation makes it wider absorption and higher ^1^O_2_ generation efficiency. Finally, the photosensitizer was further optimized to obtain polymer CPTF with a large photooxidation-specific surface area and good recyclability. The ISC channels of DTF and the two conjugated polymers increased and the ^1^O_2_ generation capacity was thus enhanced after polymerization, giving the results that the ^1^O_2_ generation efficiency of PTF was 3.63 times than that of DTP, and CPTF was 4 times than that of DTP. Meanwhile, CPTF possessed a relatively large Brunauer-Emmet-Teller (BET) surface area (117.2 m^3^g^−1^), which could be used as a photooxidation catalyst for generating ^1^O_2_ under natural sunlight or simulated AM1.5G irradiation, to oxidize benzaldehyde to benzoic acid in solvent-free conditions. In addition, the excellent photostability and poor solubility of CPTF make it easy to be separated and recycled after the reaction. In addition, due to the excellent photostability and poor solubility of CPTF, it is easy to separate and recycle after the reaction (Figure 13b). In sunlight-induced CPTF-catalyzed wastewater treatment experiments, both Rhodamine 6G and *S. aureus* were efficiently decomposed after 2 h irradiation with AM 1.5G (Figure 13c,d), and CPTF could be recovered by filtration for further use.

**Figure 14:**
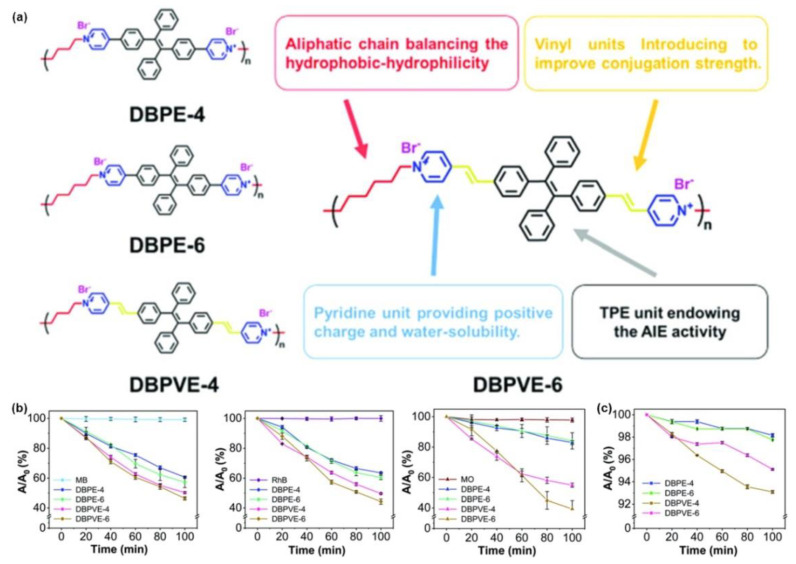
(**a**) The chemical structures of DBPE-4, DBPE-6, DBPVE-4 and DBPVE-6. (**b**) Decomposition ratio of MB, RhB and MO upon mixing with DBPE-4, DBPE-6, DBPVE-4 and DBPVE-6 under white light irradiation, respectively. (**c**) The self-decomposition rates of the four polymers under white light irradiation. Reprinted with the permission from Ref. [60]. 2021, Royal Society of Chemistry.

Following this, Xu et al. [60] prepared four AIE-active conjugated polymers, DBPEs (DBPE-4, and DBPE-6) and DBPVEs (DBPVE-4, and DBPVE-6), with different conjugation strengths and aliphatic chain lengths. Taking RB as a reference, compared with the other three polymers, DBPVE-6 with long aliphatic chains possesses the highest singlet oxygen quantum yield (0.46, the highest for DBPVE-6, 0.13 for DBPE-4, 0.14 for DBPE-6, and 0.34 for DBPVE-4). Density functional theory (DFT) simulation indicated that the higher rotational energy barrier caused by the longer aliphatic chain units has effectively limited the intramolecular rotation, thereby suppressing nonradiative transitions, showing more pronounced radiative signals during the aggregation process. Meanwhile, the relatively lower ∆*E*_ST_ value of DBPVEs also promoted the ISC process and enhanced the ^1^O_2_ quantum yield. Subsequently, three organic dyes, methylene blue (MB), rhodamine B (RhB), and methyl orange (MO), were used as wastewater treatment models to evaluate their ability to act as photosensitizers for photocatalytic degradation of wastewater. As shown in Figure 14b, benefiting from the higher ^1^O_2_ quantum yield, DBPVEs exhibited higher dye decomposition efficiency than that of DBPEs. After 120 min of illumination, DBPVE-6 showed the best photocatalytic performance in degrading organic pollutants in wastewater with decomposition efficiencies of 54.1% for MB, 56.2% for RhB, and 60.4% for MO, respectively.

## 5. Polymerization-Enhanced Room-Temperature Phosphorescence for Security Protection

**Figure 15:**
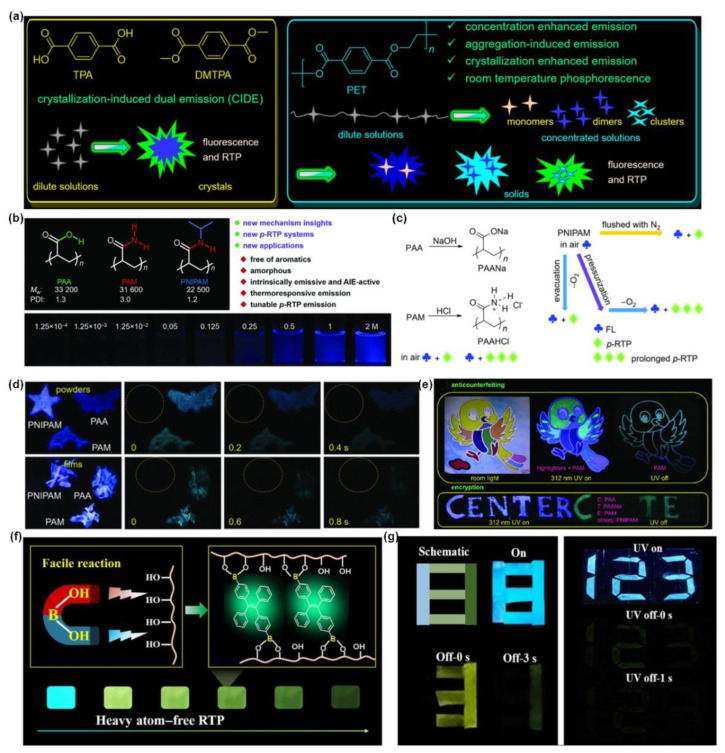
(**a**) The chemical structures of TPA, DMTPA, and PET and the schematic illustration of their emission photophysical properties in different states. Reprinted with the permission from Ref. [99]. 2018, American Chemical Society. (**b**) The chemical structures of PAA, PAM, PNIPAM and photographs of different PNIPAM/DMF solutions under 365 nm UV light. (**c**) Schematic diagram of the modulation of p-RTP properties of different polymers. (**d**) Photographs of different polymer powders taken under or after removing the 312 nm UV irradiation under nitrogen or in vacuum. (**e**) Photographs of practical application in graphic security and information encryption made from different polymers. Reprinted with the permission from Ref. [100]. 2019, Royal Society of Chemistry. (**f**) Schematic illustration of polymer-based RTP through B—O click reaction. (**g**) Photographs of practical application in data encryption, and digital coding by TPEDB ink on PVA under after removing UV irradiation. Reprinted with permission from open access of Ref. [101], 2020, American Association for the Advancement of Science.

In addition to generating ROS, triplet excitons can also be used to produce phosphorescence [26]. Room-temperature phosphorescent materials are flourishing in various fields such as anti-counterfeiting and imaging due to their long emission lifetime and rich excited-state features [86,87,88,89,90,91,92,93,94]. According to the corresponding lifetime of different materials, the information can be encrypted or decrypted by the technology of time resolution. An et al. [95] designed a series of long-life RTP molecules CzPX and CzBX (X=Cl, Br) with different lifetimes through structural modulation, and realized the related applications of information encryption. Recently, a series of representative studies have been reported by An [89,96,97]. It is found that many polymers have the ability to produce RTP by providing a rigid environment or inhibiting the movement of phosphor molecules [98]. Yuan et al. [99] systematically studied the photophysical properties of poly (ethyl terephthalate) (PET), a polymer analogue of terephthalic acid (TPA) and dimethyl terephthalate (DMTPA) with crystal-induced double emission (Figure 15a). It shows weak luminescence at low concentrations, but strong blue light emission at high concentrations and solid state, exhibiting the typical AIE phenomenon. At the same time, it shows dual fluorescence-phosphorescence emission in the solid state, and the film efficiency increases with the enhanced crystallinity accompanied by the obvious RTP phenomenon. It provides a new strategy for the molecular design of chemical sensing to monitor its own crystallization process. Similarly, Yuan et al. [100] also synthesized three amorphous polymers, all of which showed strong blue light emission in the aggregated state (Figure 15b), where fine modulation of RTP can be achieved by varying intermolecular interactions and changes in pendants. Among them, PAA and PAM solids show obvious RTP phenomenon in air conditions, and can be significantly enhanced by ionization, while the RTP of PNIPAM is quenched by oxygen, and appeared only oxygen is isolated (Figure 15c,d). Based on this, as shown in Figure 15e, the bird was colored with different commercial highlighter and PAM in the corresponding parts showing completely different emission color under 312 nm UV light. After the light source was removed, only the bird skeleton coated with PAM was visible to the naked eye. In addition, writing “CENTER” with PAA, PAM, PNIPAM and PAAN powder obtained by neutralization with NaOH, only the green “CTE” is visible under the irradiation of 312 nm UV light due to the quenching effect of oxygen. All of these have enabled the application of multi-modal anti-counterfeiting, and the unique RTP phenomenon of this amorphous non-aromatic polymer provides new ideas for the design of new cryptography materials.

Lu et al. [101] performed a simple one-step B-O click reaction of boric acid-modified tetraphenylene phosphor TPEDB with polyhydroxy PVA matrix to immobilize TPEDB in a polymer network through covalent bonding for rapid preparation of RTP materials, and their RTP performance can be well tuned by adjusting the number of B-O bonds (Figure 15f). The theoretical calculations show that the PVA matrix provides a closed microenvironment in which the TPEDB phosphorescence is effectively immobilized, inhibiting its non-radiative transition path and thus activating RTP emission. Subsequently, the practicality of the TPEDB-PVA polymer material for use in cryptography has subsequently been explored. As shown in Figure 15g, the number “8” was structurally split and encoded using TPEDB-PVA materials with varying alcoholysis degrees of PVA, showing bright blue emission under UV light. After the light source is removed, different afterglow durations of the corresponding materials can exhibit different numbers.

## 6. Conclusions and Perspectives

The design and regulation of high-performance luminescent materials have always been hotspot research in various frontier fields. In addition to widely used small molecules, luminescent polymers are designed based on fluorescent small molecules through polymerization reactions or modification by grafting small molecule fluorophores onto side chains of polymer structure. Combined with the advantages, such as good thermal stability and processability of polymers themselves, luminescent polymers have occupied an essential position in wide fields. As an alternative to traditional light-emitting materials, AIE materials have been extensively studied and expanded since they were reported due to their excellent photophysical properties such as enhanced fluorescence in the aggregated state. Among the whole luminescent material’s family, AIE-active polymers, blending with both characteristics of polymers and AIEgens, have achieved important progress and breakthroughs in the research of high-performance optical materials since they were first reported in 2003, especially in recent years.

Based on numerous successful examples, in this review, recent research progress in enhancing several optical properties of AIE materials through polymerization, including luminescence intensity, ROS production capability, photocatalysis, RTP etc. has been reviewed in detail. In these sections, polymerization plays a vitally important role for improving various optical properties by one or more of the following ways: (1) Participating in the reaction as polymerization initiator, as the reaction progresses, the viscosity of the system gradually increased, along with the enhancement of luminescence intensity, and thus the degree of polymerization could be monitored and tracked through a visual method. (2) Increasing the number of repeating units, extending the conjugation length, and reducing the value of singlet-triplet energy gap ∆*E*_ST_, thereby promoting the ISC process and enhancing the efficiency of ROS generation. (3) Increasing the rotational barrier and suppressing the intramolecular rotation to reduce the energy dissipation caused by non-radiative transitions. (4) Producing RTP by providing a rigid environment or inhibiting the movement of phosphor molecules, etc. Although some discussions on the results have been verified or presented by means of tests and theoretical calculations, more and more comprehensive examples are still needed to verify the generality of polymerization in enhancing the optical properties of AIE materials, so as to fully meet the needs of various fields in the future. In addition to the cases cited above, examples of AIE-active polymers for photothermal therapy applications have also been reported. We believe that with the deepening of research, more optical properties improved or enhanced by polymerization will be reported, and further research will be conducted to achieve greater breakthroughs.

## Data Availability

No Applicable.
